# Different Effect of *Sox11* in Retinal Ganglion Cells Survival and Axon Regeneration

**DOI:** 10.3389/fgene.2018.00633

**Published:** 2018-12-18

**Authors:** Ying Li, Felix L. Struebing, Jiaxing Wang, Rebecca King, Eldon E. Geisert

**Affiliations:** ^1^Department of Ophthalmology, Emory University, Atlanta, GA, United States; ^2^Center for Neuropathology and Prion Research, Ludwig Maximilian University of Munich, Munich, Germany; ^3^Department for Translational Brain Research, German Center for Neurodegenerative Diseases, Munich, Germany

**Keywords:** retinal ganglion cells, *Sox11*, optic nerve crush, axon regeneration, AAV2

## Abstract

**Purpose:** The present study examines the role of *Sox11* in the initial response of retinal ganglion cells (RGCs) to axon damage and in optic nerve regeneration in mouse.

**Methods:** Markers of retinal injury were identified using the normal retina database and optic nerve crush (ONC) database on GeneNetwork^2^ (www.genenetwork.org). One gene, *Sox11*, was highly upregulated following ONC. We examined the role of this transcription factor, *Sox11*, following ONC and optic nerve regeneration in mice. *In situ* hybridization was performed using the Affymetrix 2-plex Quantigene View RNA *In Situ* Hybridization Tissue Assay System. *Sox11* was partially knocked out by intravitreal injection of AAV2-CMV-Cre-GFP in *Sox11*^f/f^ mice. Optic nerve regeneration model used *Pten* knockdown. Mice were perfused and the retinas and optic nerves were dissected and examined for RGC survival and axon growth.

**Results:** *Sox11* was dramatically upregulated in the retina following ONC injury. The level of *Sox11* message increased by approximately eightfold 2 days after ONC. *In situ* hybridization demonstrated low-level *Sox11* message in RGCs and cells in the inner nuclear layer in the normal retina as well as a profound increase in *Sox11* message within the ganglion cells following ONC. In *Sox11*^f/f^ retinas, partially knocking out *Sox11* significantly increased RGC survival after ONC as compared to the AAV2-CMV-GFP control group; however, it had little effect on the ability of axon regeneration. Combinatorial downregulation of both *Sox11* and *Pten* resulted in a significant increase in RGC survival as compared to *Pten* knockdown only. When *Pten* was knocked down there was a remarkable increase in the number and the length of regenerating axons. Partially knocking out *Sox11* in combination with *Pten* deletion resulted in a fewer regenerating axons.

**Conclusion:** Taken together, these data demonstrate that *Sox11* is involved in the initial response of the retina to injury, playing a role in the early attempts of axon regeneration and neuronal survival. Downregulation of *Sox11* aids in RGC survival following injury of optic nerve axons, while a partial knockout of *Sox11* negates the axon regeneration stimulated by *Pten* knockdown.

## Introduction

Advances in our ability to monitor molecular changes in neurons have led to an increased understanding of the events that transpire following neuronal injury (Howell et al., 2011; Moore and Goldberg, 2011; Vazquez-Chona and Geisert, 2012; Munguba et al., 2013; Templeton et al., 2013). After axonal damage, the initial response of a central nervous system (CNS) neuron may be similar to that of a neuron in the peripheral nervous system (PNS) (Carlstedt, 1997). Ultimately, neurons in the PNS will regenerate their axons and survive, while those in the CNS do not regenerate their axons and the cell bodies die. The initial response of the CNS neurons to regenerate and eventual failure of this regenerative response was described by Ramón y Cajal and termed “abortive regeneration” (Ramón et al., 1928). When examining the initial response of neurons to injury, there are some common responses in the CNS and PNS. One transcription factor activated in both the CNS and PNS after injury is *Sox11* (McCurley and Callard, 2010; Struebing et al., 2017). There is strong evidence that this gene is part of the transcriptional network activated by injury and involved in axonal regeneration in the PNS (Jankowski et al., 2009; Jing et al., 2012).

*Sox11* is a member of the SRY-related box group C (*SoxC*) gene family of transcription factors (Schepers et al., 2002; Pillai-Kastoori et al., 2015). Among these, SOX11 and SOX4 play a critical role in the normal development of neurons and specifically retinal ganglion cells (RGCs) (Jiang et al., 2013; Usui et al., 2013a; Wang et al., 2013). SOX11 is expressed in retinal progenitor cells as part of the process leading progenitor cells to become neuroblasts (Haslinger et al., 2009; Usui et al., 2013b). In knockdown of either *Sox11* or *Sox4*, there is a moderate reduction in RGC number; however when both *Sox11* and *Sox4* are knocked down, there is a complete loss of ganglion cell development (Jiang et al., 2013). During eye development, *Sox11* is also required to maintain proper levels of *hedgehog* signaling, and mutations have been associated with coloboma due to improper optic fissure closure (Pillai-Kastoori et al., 2014; Wen et al., 2015). Furthermore, SOX11 is critical for axonal growth, driving the expression of axon growth-related proteins such as class III beta tubulin and MAP2 (Bergsland et al., 2006). SOX11 also plays a similar role in adult neurogenesis. High levels of SOX11 are found in the cells within the subventricular zone, the rostral migratory stream and within the neuroprogenitor zone of the dentate gyrus (Tanaka et al., 2004; Haslinger et al., 2009; Wang et al., 2013). These studies underline the importance of SOX11 in terminal differentiation of progenitor cells to neurons and axon extension.

In addition to functioning in neuronal differentiation, SOX11 has a prominent role in the response of neurons to injury. After peripheral nerve injury, SOX11 is immediately upregulated in the neuronal cell bodies as the axon is regenerating (Tanabe et al., 2003; Jankowski et al., 2009). Decreasing levels of SOX11 in the neuronal cell body result in slower axonal regeneration of peripheral nerves (Jankowski et al., 2009). Similar results are observed in tissue culture. When *Sox11* is knocked down in cultured peripheral neurons, there is also a reduction in neurite growth and an increase in apoptosis (Jankowski et al., 2006). Conversely, overexpressing *Sox11* in cultured dorsal root ganglion cells produces an increase in neurite growth, and *in vivo* overexpression of *Sox11* accelerates the growth of regenerating axons (Jing et al., 2012). One intriguing anatomical experimental model is the dorsal root ganglion, where the central projection of the dorsal root ganglion enters the spinal cord (CNS) and the peripheral projection extends out into a peripheral nerve that is myelinated by Schwann cells. When the central rootlet is severed, there is a modest (51%) increase in *Sox11* expression in the ganglion even when the central portion will not regenerate back into the spinal cord. However, when the peripheral root is damaged, a relatively massive (1004%) increase in *Sox11* is seen as the axons regenerate down the peripheral nerve (Jankowski et al., 2009).

In the present study, we examine the response of SOX11 in the retina following injuries to the axons of the optic nerve. We also explored the potential role of *Sox11* in injured RGCs and regenerated axons. We propose that the upregulation of SOX11 after injury is an attempt of neurons to regenerate, but ultimately results in abortive regeneration and cell death.

## Materials and Methods

### Mice

All procedures involving animals were approved by the Animal Care and Use Committee of Emory University and were in accordance with the ARVO Statement for the Use of Animals in Ophthalmic and Vision Research. BXD stains, including their parental strains, C57BL/6J and DBA/2J, were used for Gene network database. *Sox11*^f/f^ mice used for *Sox11* downregulation experiment and the regeneration study were obtained from Dr. Rafi Ahmed’s labs, which were originally created by Veronique Lefebvre at Cleveland Clinic (Bhattaram et al., 2010). The mice were housed in a pathogen-free facility at Emory University, maintained on a 12 h:12 h light–dark cycle, and provided with food and water *ad libitum.*

### Optic Nerve Crush

The optic nerve crush (ONC) procedure was performed as previously described in Templeton and Geisert (Templeton and Geisert, 2012). Briefly, C57BL/6 or *Sox11*^f/f^ (four mice per group) mice were deeply anesthetized with 15 mg/kg xylazine and 100 mg/kg ketamine for the surgery. An incision was made into the lateral aspect of the conjunctiva, and the eye was rotated nasally to expose the optic nerve. The optic nerve was then grasped for 10 s with Dumont cross-clamp #7 forceps (Roboz, cat. #RS = 5027, Gaithersburg, MD, United States), using only the spring action of the instrument to crush the nerve. The crush site is about 1 mm behind the eye to avoid injury to the ophthalmic artery as mentioned by Wang et al. (2018). The animals were allowed to recover on a water-circulated heating pad.

### *In situ* Hybridization

*In situ* hybridization was performed using the 2-plex Quantigene View RNA ISH Tissue Assay kit (Affymetrix, Inc., Santa Clara, CA, United States). Assays were performed as per the manufacturer’s instructions with stock solutions. Eyes were isolated from C57BL/6 mice (*n* = 6), both control and 2 days after ONC, and drop-fixed in 4% Paraformaldehyde for 24 h. Mid-way through the 24-h fixation, a 26-gauge needle was used to create a hole in the cornea, assisting with the fixation. Immediately after the 24-h period, the eyes were serially dehydrated in ethanol, cleared with xylenes embedded in paraffin. Blocks were sectioned on an American Optical series 1000 microtome (American Optical, Co., Buffalo, NY, United States) to a thickness of 5 μm and mounted on Surgipath X-tra micro slides (Leica Biosystems Richmond, Inc., Richmond, IL, United States). Before beginning the hybridization protocol, the slides were baked at 60°C for 30 min to increase tissue adhesion. As per the manufacturer’s protocol, the paraffin was removed from the slides with xylene before being boiled in a pretreatment solution (Affymetrix) for 10 min and incubated with Protein Kinase K (Affymetrix, Santa Clara, CA, United States) at 40°C for 10 min. Custom probes for *Sox11* and *Chrna6* (Affymetrix, Santa Clara, CA, United States) were then hybridized to the tissue. Signal amplification was accomplished by hybridizing Type 1 and Type 2 specific pre-amp oligonucleotides, amp oligonucleotides and label oligonucleotides sequentially, achieving a 400-fold signal amplification from each mRNA molecule. Sections were viewed with an Olympus BX51 microscope (Olympus America, Inc., Melville, NY, United States).

### *Sox11* Partial Knockout

**FIGURE 1:**
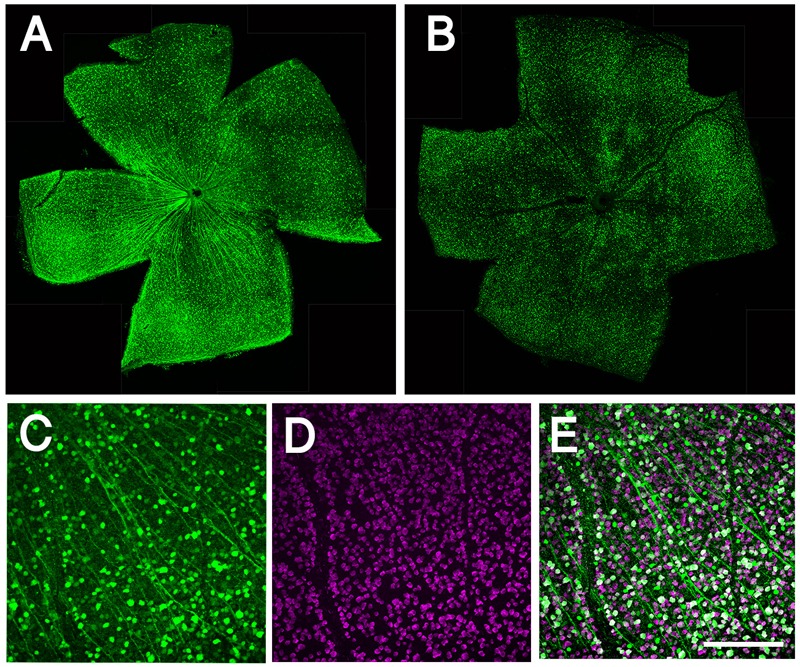
Whole mount retinal immunohistochemistry showing AAV transduction in RGCs. **(A)** Confocal image of whole mounted retina showing transduction of AAV2-CMV-GFP. **(B)** Confocal image of whole mounted retina showing transduction of AAV2-shRNA- *Pten*-GFP. **(C)** Confocal image of flat mounted retina showing GFP expressing in RGCs. **(D)** RBPMS label RGCs specifically as a marker. **(E)** More than half of RGCs are transduced by AAV and express GFP; merge image. Scale bar = 100 μm.

To downregulate *Sox11, Sox11*^f/f^ mice received intravitreal injections of AAV2-CMV-GFP-Cre [2 μl of 10^12^ vg/mL, expressing Cre recombinase with GFP from a bicistronic vector (Addgene plasmid #49056), produced by Fred Gage (Kaspar et al., 2002)]. For the control animals, a vector expressing only GFP (AAV2-GFP under the CMV promoter) was used. Two weeks following intravitreal injection, optic nerves were crushed. For analysis of transduction efficiency, we included four injected animals (four eyes). Images from five randomly selected fields of each retina were taken at 20x magnification. The average transduction efficiency of AAV2 vector was 54%, based on the number of GFP-positive and RBPMS-positive cells in flat-mount retinas (Figure 1).

### *Pten* Deletion Induced Regeneration Model and Quantitation of Axon Regeneration

We used AAV2-shRNA-*Pten*-GFP (*Pten* short hairpin RNA-GFP packaged into AAV_2_ backbone constructs, titer = 1.5 × 10^12^ vg/ml) for *Pten* knockdown. The shRNA target sequence is 5′-AGG TGA AGA TAT ATT CCT CCA A-3′ as described by Zukor et al. (2013). Deletion of Pten is showed to promote robust axon regeneration after ONC (Park et al., 2008). Two weeks before ONC, the mice were intravitreally injected with 2 μl AAV-shRNA-*Pten*-GFP. Optic crush was performed. Zymosan (Sigma, St. Louis, MO, United States; Z4250, Lot # BCBQ8437V) along with cAMP analog CPT-cAMP (Sigma, C3912, Lot # SLBH5204V) total volume 2 μl were injected into vitreous immediately following ONC to induce an inflammatory response and augment regeneration. Twelve days after ONC (2 days before euthanasia), Alexa Fluor^®^ 647-conjugated Cholera Toxin B (CTB; Thermo Fisher, Waltham, MA, United States; C34778) was injected into the vitreous for anterograde labeling of the regenerated axons. At 14 days after ONC, the mice were deeply anesthetized with a mixture of 15 mg/kg of xylazine and 100 mg/kg of ketamine and perfused through the heart with PBS (Phosphate Buffered Saline, diluted to 1x from 10x stock, Corning, Manassas, VA, United States, pH 7.3) followed by 4% paraformaldehyde in phosphate buffer (pH 7.3).

### Immunohistochemistry

For the retina whole mount, retina tissues were removed from the globe after deeply anesthesia and perfusion with 4% paraformaldehyde, and fixed in 4% paraformaldehyde at room temperature for 1 h. Following post-fixation, intact retinas were extracted and washed in PBST (Phosphate Buffered Saline with Tween 20) with 0.5% Triton X-100. Retinas were blocked in 5% BSA (Sigma) and 10% donkey serum in PBS for 2 h followed by incubation with primary antibodies for 24 h at 4°C. The following primary antibodies and dilutions were used: anti-GFP (Novus Biologicals, Littleton, CO, United States; Cat. # NB100–1770, 1:1000), anti-RBPMS (Millipore, Cat # ABN1376, Burlington, MA, United States, 1:1000). Then retinas were washed in PBST three times 10 min each, and incubated in secondary antibodies in PBST overnight at 4°C. After another triplicate wash, retinas were carefully flattened and mounted on microscopy slides with Fluoromount (Southern Biotech, Birmingham, AL, United States) and imaged using a Nikon Eclipse Ti (Nikon, Inc., Melville, NY, United States) confocal microscope.

### Counting Surviving RGCs and Regenerating Axons for Statistical Analysis

Retinal flat-mounts were immunostained with RBPMS antibody (Millipore, Cat # ABN1376, Burlington, MA, United States) to label RGCs. For each retina, five fields, whose distance to the optic nerve were equal, were randomly sampled from middle regions of each retina under 20x magnification. The density of RGCs per field was calculated according to the number of RGCs per field. The GFP and RBPMS double-labeled cells were counted as well, as the transduced cell results.

Optic nerves along with the optic chiasm were dissected and post-fixed in 4% paraformaldehyde overnight. The optic nerve was cleared with FocusClear^TM^ (CelExplorer, Hsinchu, Taiwan) until totally transparent. FocusClear allows us to scan the whole thickness of the optic nerve for better understanding of the status of axon regeneration and provide clear imaging of regenerated axons from the optical slices scanned by confocal microscope for counting. The quantitation of axon regeneration was determined by counting the longest five axons or the longest single axon along the whole nerve. Both the distance the axons traveled and the number of axons at 0.5 and 1 mm behind the crush site were measured as described in Wang et al. (2018). Pseudocolor of green was used to show the CTB-labeled axons in all the optic nerve images of this study for clear visual observation. Data are presented as mean ± standard error of the mean (SEM). Differences in axon counts, regeneration distance, and transduction efficiency were analyzed with the Mann–Whitney *U*-test using SPSS Statistics package 24.0 (SPSS, IBM, Chicago, IL, United States). A *p*-value of less than 0.05 was considered statistically significant.

All the experiments were done following standard biosecurity and institutional safety procedures.

## Results

### The Distinct Expression Changes of *Sox11* Following Injuries to the Axons of the Optic Nerve

**FIGURE 2:**
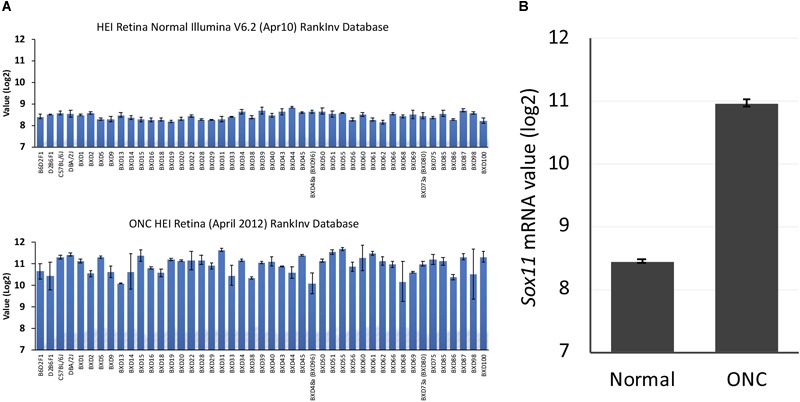
*Sox11* expression in the normal retina and after optic nerve crush (ONC). **(A)** The ordinate represents mRNA levels from microarrays, expressed in a log_2_ scale with the mean set to 8. The mice used to generate these data were C57BL/6, DBA/2J, their respective F1 crosses, BALB/cByJ, and members of the BXD recombinant inbred strain line. The top graph denotes the expression levels of *Sox11* under normal conditions, while the bottom graph denotes the expression of *Sox11* 2 days after the ONC. **(B)** Note the dramatic increase in expression following injury, from a normal mean expression value of 8.4 to a mean expression value of 11.0 after nerve crush, which corresponded to an approximately eightfold increase.

To define the changes in *Sox11* that occur following injury to the optic nerve, we compared *Sox11* mRNA levels in normal mice to *Sox11* levels following ONC using the bioinformatic tools on GeneNetwork^1^. The levels of *Sox11* mRNA were examined using the Normal HEI Retina (April, 2010) Database and comparing it to the ONC HEI Retina (April, 2012) Database (Freeman et al., 2011; Templeton et al., 2013). *Sox11* was one of the genes with the largest change in expression 2 days after ONC. In the normal retinal dataset, the mean expression for *Sox11* (detected by Illumina probe ILMN_1235647) across the BXD RI strains was 8.4 on a log_2_ Z-scale (The mRNA expression levels are expressed on a custom z-scale according to the formula 2Z + 8 so that the mean expression of all mRNAs on the array equals 8) (Parker et al., 2017). In the C57BL/6 parental strain, the expression level in the normal retina was 8.59 and for the DBA/2J strain the mean expression level was 8.54. Two days after ONC, there was a dramatic increase in the level of *Sox11* expression (Figure 2), with the mean expression across the BXD strains being 11.03, which corresponded to an approximately eightfold increase. The same increase was observed in individual strains. The C57BL/6 strain had an expression level of 11.33 after ONC and the expression in the DBA/2J strain increased to 11.44. These data indicate that *Sox11* is dramatically upregulated after a specific injury to the ganglion cell axons within the optic nerve.

### Expression Pattern of *Sox11* in Naïve Adult Retina and Injured Adult Retina

**FIGURE 3:**
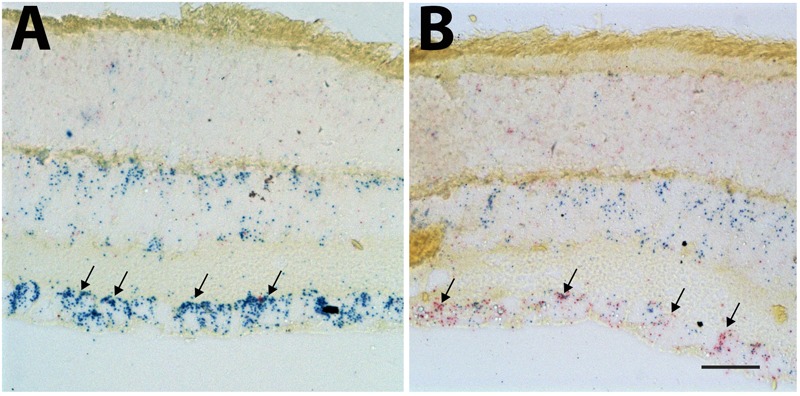
*In situ* hybridization for *Sox11* and *Chrna6.* The distribution of *Sox11* and *Chrna6* mRNA was defined using two-color *in situ* hybridization for a section of control retina **(A)** and a section of a retina 2 days after ONC **(B)**. The probe for *Chrna6* is shown in blue and that for *Sox11* in red. Note the extensive staining for *Chrna6* within the ganglion cell layer in the control section (Arrows in **A**). Two days after ONC there is already a substantial decrease in *Chrna6* expression. Very low levels of *Sox11* are seen in the control retina. There is a dramatic increase in *Sox11* staining after nerve crush within the retinal ganglion cells (RGCs) (Arrows in **B**). **(A,B)** Are taken at the same magnification and the scale bar in **(B)** = 25 μm.

*In situ* hybridization was used to identify the cells expressing *Sox11* after injury to the retina. For the *in situ* hybridization, we examined retinas 2 days after ONC (Figure 3B) and compared it to uninjured control retinas (Figure 3A). Using the Affymetrix 2-plex Quantigene View RNA ISH Tissue Assay kit, we labeled cells expressing the RGC marker *Chrna6* blue and *Sox11* red (Figure 3) (Munguba et al., 2013). In the control retina, many of the cells in the ganglion cell layer were heavily labeled for *Chrna6*, and a few of the cells expressed marginal levels of *Sox11*. Two days after ONC there was a dramatic decrease in the labeling for *Chrna6* and a substantial increase in the amount of labeling for *Sox11*. This change was similar trend to the changes in message levels observed in our microarray databases on GeneNetwork.org. In the normal retina database, *Chrna6* (probe ILMN_2732438) was expressed at relatively high levels (mean value of 11.04) and 2 days following ONC, the expression decreased twofold (mean value of 9.96). These data from the *in situ* hybridization mirror our microarray expression analysis results.

### Downregulation of *Sox11* Facilitates RGC Survival Following Optic Nerve Crush

**FIGURE 4:**
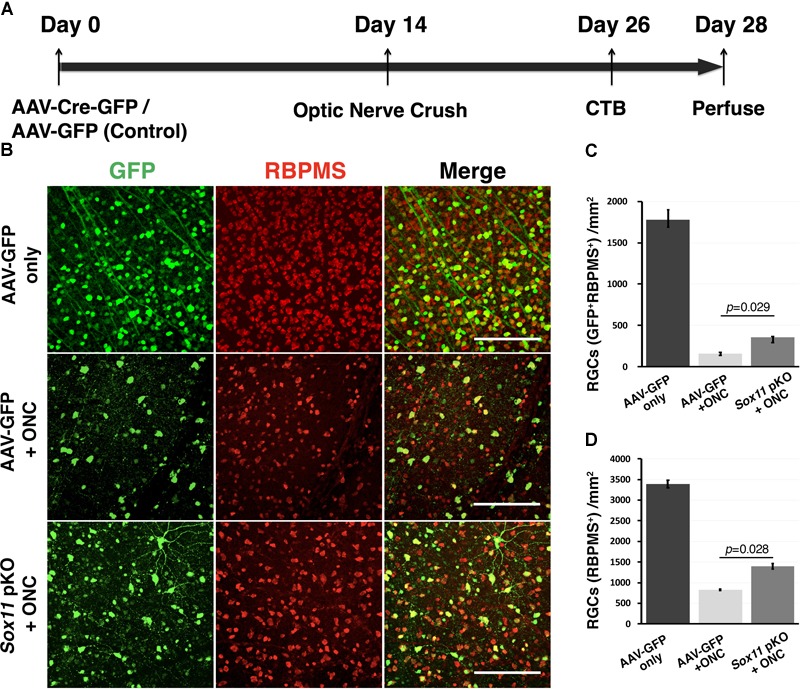
Downregulation of *Sox11* facilitates RGC survival following ONC. **(A)** *Sox11* is partially knocked out by intravitreal injection of AAV2-CMV-Cre-GFP in 4 to 6-week-old *Sox11*^f/f^ mice. The control group receives AAV2-CMV-GFP. Optic nerve crush is performed 2 weeks after viral vector injection. Twelve days after ONC, Alexa Fluor 647-conjugated cholera toxin B (CTB) is used to label regenerating axons. Two days later mice are sacrificed. **(B)** For the retinal whole mounts, RGCs are labeled with the pan-RGC marker RBPMS. The number of AAV2-GFP vector transduced RGCs without crush injury is 1782 ± 117.5/mm^2^. **(C)** Downregulation of *Sox11* in RGCs greatly increases the survival of AAV2 vector transduced RGCs (double labeled by GFP and RBPMS) after ONC as compared to AAV2-CMV-GFP injection with ONC group (357 ± 7.1/mm^2^ vs. 158 ± 15.7/mm^2^, *n* = 4, *p* = 0.029, Mann–Whitney *U*-test). **(D)** The number of total RGCs labeled by RBPMS without crush injury is 3389 ± 92.1/mm^2^. When comparing the total RGCs labeled by RBPMS, it shows that *Sox11* partial knockout in the *Sox11*^f/f^ mice significantly increases RPBMS-positive RGC survival after ONC as compared to AAV2-CMV-GFP ONC group (1398 ± 63/mm^2^ vs. 828 ± 23/mm^2^, *n* = 4, *p* = 0.028, Mann–Whitney *U*-test). Scale bar = 100 μm.

To define the role of *Sox11* in the response of RGC to Injury, an AAV2 vector expressing Cre and GFP was used for *Sox11* partial knockout in RGCs of the *Sox11*^f/f^ mice (Figure 4). The retinas containing *Sox11* partial knockout cells were compared to retinas treated in a similar manner using AAV2-GFP vectors. Two weeks after the intravitreal injection of the AAV2-Cre-GFP or AAV2-GFP vectors, the optic nerve was crushed. Following a 26-day survival, the eyes were injected with fluorescence-labeled CTB and the animals were sacrificed 2 days later. These data were compared to control retinas injected with AAV2-GFP vector that did not receive a nerve crush (Figure 4B). The flat mounted retinas were immunostained for RBPMS to label RGCs. When examining the retinas, we found an increased number of GFP-labeled RGCs in the *Sox11* partial knockout animals relative to the GFP control animals (Figures 4B,C). It is also the case for all of the RBPMS labeled RGCs (Figures 4B,D). There were fewer RGCs left in the GFP control retinas relative to the retinas where *Sox11* was partially knocked out. These results were quantified, counting the number of GFP-labeled RGCs (double labeled by GFP and RBPMS, Figure 4C) and total RGCs (labeled by RBPMS, Figure 4D) to define the effects of partially knocking out *Sox11*. The normal retinas (without ONC) had 1782 GFP-labeled RGCs/mm^2^ (SE 130 Cells/mm^2^, *n* = 4). In the crushed retinas that received AAV2-GFP, the average number of GFP-labeled RGCs was 158/mm^2^ (SE 15.7 Cells/mm^2^, *n* = 4); while, in the retinas receiving AAV2-Cre-GFP, the average number of GFP-labeled RGCs 357 RGCs/mm^2^ (SE 7.1 Cells/mm^2^, *n* = 4). This 125.9% increase in RGC survival between AAV2-GFP group and AAV2-Cre-GFP group is statistically significant (*p* = 0.029, Mann–Whitney *U*-test, *n* = 4, Figure 4C). Interestingly, if we examine the number of RBPMS-labeled RGCs in the same retinas (Figure 4D), with a dramatic decrease caused by the ONC, a similar protective effect observed in the retinas in which *Sox11* was knocked down using AAV2-Cre-GFP (Figure 4D). Without ONC, the retinas had 3389 RBPMS-labeled RGCs/mm^2^ (SE 92.1 Cells/mm^2^, *n* = 4). In the crushed retinas that received AAV2-GFP, the average number of total RGCs was 828/mm^2^ (SE 22.9 Cells/mm^2^, *n* = 4); while, in the retinas receiving AAV2-Cre-GFP, the average number of total RGCs 1398 RGCs/mm^2^ (SE 62.9 Cells/mm^2^, *n* = 4). Partial knockout *Sox11* had a significantly positive effect on the survival of the RGC cell body 14 days after ONC (*p* = 0.028, Mann–Whitney *U*-test, *n* = 4). Thus, a partial knockout of *Sox11* not only rescued the transduced cells missing *Sox11* but there was also a significant increase in survival of non-transduced RGCs in retinas.

### *Sox11* Partial Knockout Does Not Facilitate Axon Regeneration

**FIGURE 5:**
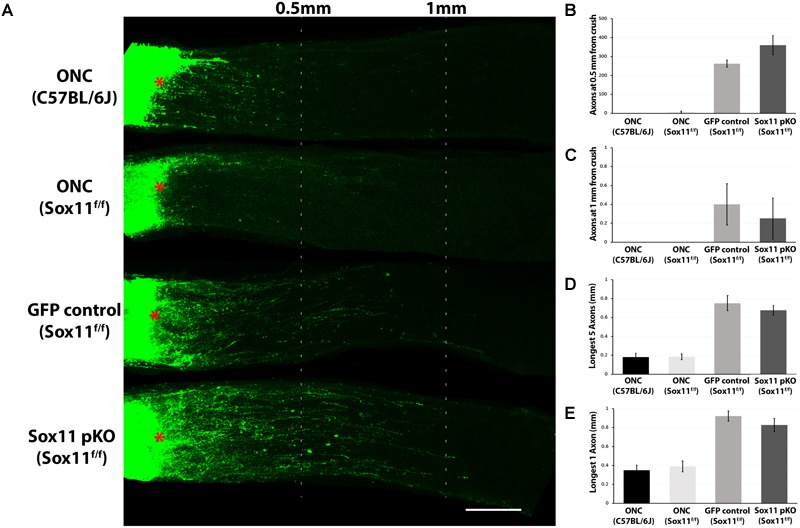
Representative images of optic nerves showing regenerating axons from different groups at 2 weeks after injury. **(A)** Pseudocolor green was used for the CTB-labeled axons in all the optic nerve images of this study for clear visual observation (red asterisks represent the crush site). Quantification axon number at 0.5 mm **(B)** and 1 mm **(C)** from crush site reveals that there is a statistically significant increase in the number of axons following an injection of either AAV2-GFP or AAV2-Cre-GFP in the *Sox11*^f/f^ mice relative to ether the C57BL/6J control of the *Sox11*^f/f^ control. This indicates that the increased axon regeneration is due to the use of AAV2-gene delivery systems in the retina and not the partial knockout of *Sox11*. A similar result was observed for the distance of longest five regenerating axons traveled **(D)** and the distance of longest single regenerating axons **(E)**. Again, the most regeneration was observed in the animals that received gene delivery in the retina by AAV2 vectors. There was a significant difference (*p* < 0.05, *n* = 4, Mann–Whitney *U*-test) between the ONC only mice and the Sox11^f/f^ mice that received intravitreal injections of either AAV2-GFP or AAV2-Cre-GFP. No statistic difference was found between the two ONC only groups. No statistic difference was found between the *Sox11* pKO and the GFP control groups. *N* = 4 per group. Scale bar = 200 μm.

When we examined the optic nerve in the same set of mice, we did not observe any significant increase in axonal regeneration that was due selectively to the partial knockout *Sox11.* We examined axon regeneration following ONC in four groups of mice: C57BL/6J controls; *Sox11*^f/f^ controls; *Sox11*^f/f^ that received an intravitreal injection of AAV2-GFP; and, *Sox11*^f/f^ that received an intravitreal injection of AAV2-Cre-GFP. The number of regenerating axons in the damaged optic nerve and the distance the axons traveled along the nerve was measured. There was very little axonal regeneration in the control groups and no statistical difference between the C57BL/6J controls and the *Sox11*^f/f^ controls. There was a significant difference (*p* < 0.05, Mann–Whitney *U*-test, *n* = 4) between these two control groups and the *Sox11*^f/f^ mice that received intravitreal injections of either AAV2-GFP or AAV2-Cre-GFP. However, there was no difference in the partial knockout of *Sox11* compared to the GFP control groups (Figure 5). These data suggest that a partial knockout of *Sox11* does not facilitate axon regeneration in the injured optic nerve. Furthermore, the AAV treatment alone (either AAV2-GFP treated *Sox11*^f/f^ mice or AAV2-Cre-GFP *Sox11*^f/f^ mice) produces a significant increase in regeneration compared to ONC only mice (non-treated C57BL/6J mice or non-treated *Sox11*^f/f^ mice). We do not fully understand this effect of AAV in the *Sox11*^f/f^ mice; however, it is a consistent result. We assume that there is some interaction between the AAV vector and the genome of the *Sox11*^f/f^ mice, facilitating axon regeneration.

### Downregulation of *Sox11* Suppresses Induced Axon Regeneration

**FIGURE 6:**
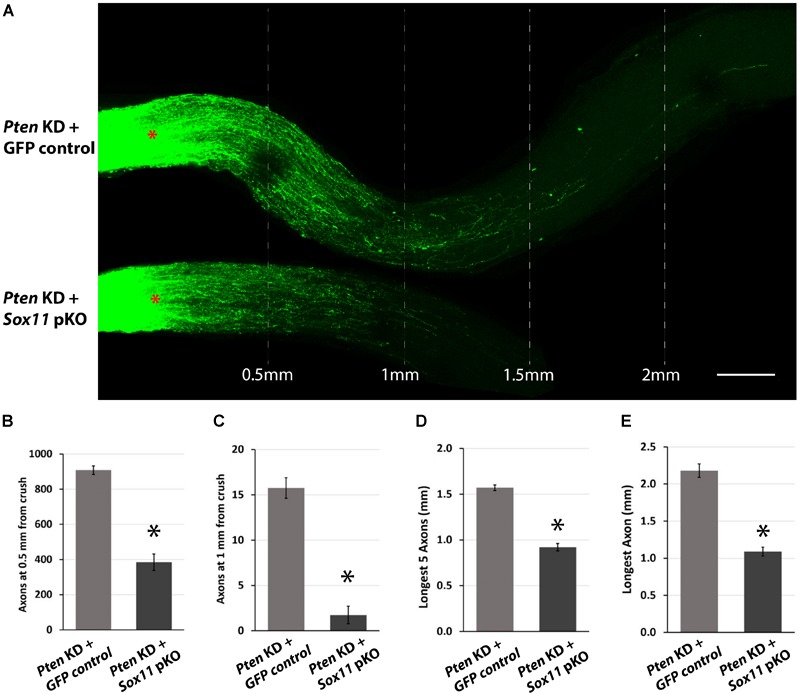
Representative images of optic nerves showing regenerating axons from different groups at 2 weeks after injury. **(A)** Pseudocolor green was used for the CTB-labeled axons in all the optic nerve images of this study for clear visual observation (red asterisks represent the crush site). Quantitative analysis of axon regeneration revealed that downregulation of *Sox11* suppresses the RGC axon regeneration following silencing of *Pten*. **(B)** The results were quantified and are displayed in a series of bar graphs showing mean and standard error of the mean in the number of regenerating axons at 0.5 mm from crush site (*n* = 4, ^∗^*p* < 0.05, Mann–Whitney *U*-test), **(C)** the number of regenerating axons at 1 mm from crush site (*n* = 4, ^∗^*p* < 0.05, Mann–Whitney *U*-test), **(D)** the longest five regenerating axons (*n* = 4, ^∗^*p* < 0.05, Mann–Whitney *U*-test), and **(E)** the longest single regenerating axon (*n* = 4, ^∗^*p* < 0.05, Mann–Whitney *U*-test). Scale bar = 200 μm.

To examine the potential contribution of *Sox11* to optic nerve axonal regeneration, a combined partial knockout of both *Sox11* and *Pten* was examined. Knocking down *Pten* with the AAV2-shRNA*Pten* vector resulted in a significant amount of axonal regeneration 2 weeks after ONC. The eyes that received AAV2-GFP and AAV2-shRNA*Pten* had a significant increase in the number of axons regenerating 0.5 mm from the crush site and an increase in the distance that longest axon traveled (Figure 6A). In the GFP control optic nerve the number of axons at 0.5 mm from the crush site was 261 ± 19 (*n* = 4) and the longest regenerating axon found in the optic nerve was on average 0.92 mm (Figure 6A). Following AAV2-shRNA*Pten*, the number of axons at 0.5 mm increased to 908 ± 24 (*n* = 4) and the longest axon was on average 2.18 mm from the crush site. When *Sox11* was partially knocked out in mice that also received the AAV2-shRNA*Pten* treatment, there was a surprising decrease in the regenerative response in the optic nerve (Figure 6A). In the *Sox11* partial knockout optic nerve the number of axons at 0.5 mm from the crush site was 384 ± 47 (*n* = 4) and the longest regenerating axon found in the optic nerve was on average 1.09 mm (Figures 6B–E). This represents a significant decrease in the number of regenerating axons (*p* = 0.02, Mann–Whitney *U-*test, *n* = 4) and the distance traveled (*p* = 0.021, Mann–Whitney *U*-test, *n* = 4).

**FIGURE 7:**
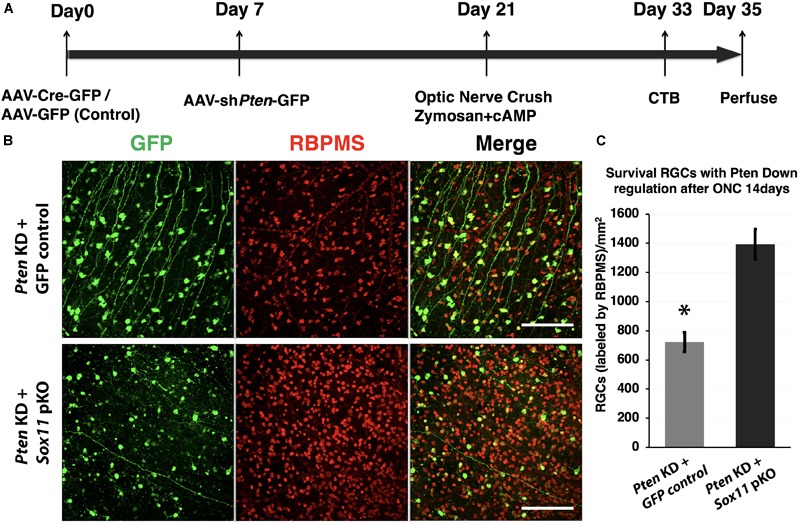
Downregulation of *Sox11* facilitates RGC survival following optic nerve crush and regeneration experiment. **(A)** *Sox11* and *Pten* are knocked down by intravitreal injection of AAV2-CMV-Cre-GFP or AAV2-shRNA-*Pten*-GFP in 4 to 6 week-old *Sox11*^f/f^ mice. The control group receives AAV2-CMV-GFP. Optic nerve crush is performed 2 weeks after viral vector injection. Twelve days after ONC, Alexa Fluor^®^ 647-conjugated CTB is used to label regenerating axons. Two days after the CTB injections the animals are sacrificed, the retinas and full-length optic nerves are removed. **(B)** RGCs are labeled with pan-RGC marker RBPMS in retinal whole mounts. **(C)** Downregulation of both *Sox11* and *Pten* resulted in a significant increase in RGC survival as compared to *Pten* knockdown only (1393 ± 94/mm^2^ vs. 722 ± 77/mm^2^, *n* = 4, ^∗^*p* < 0.05, Mann–Whitney *U*-test). Scale bar = 100 μm.

**FIGURE 8:**
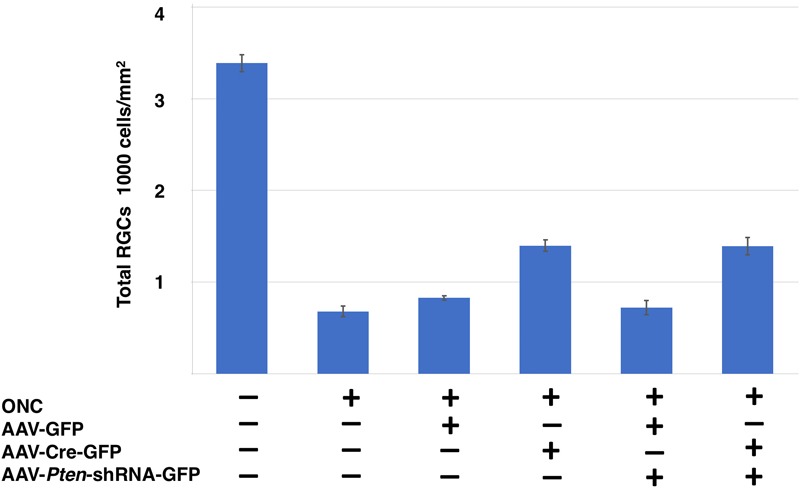
Comparison of RGCs survival following different conditions in *Sox11*^f/f^ mice. Total RGCs numbers labeled by RBPMS from six different conditions are shown as: naïve retina, crushed only retina, GFP control vector injected retina, *Sox11* partial knockout, *Pten* knockdown retina, *Pten*/*Sox11* double knockdown retina. *Sox11* deletion aids more RGCs survive after injury. *Sox11* deletion aids more RGCs survive after ONC 2 weeks.

The decrease in axonal regeneration observed in the *Pten/Sox11* double knockdown group was not due to a decrease in the number of surviving RGCs at 2 weeks following crush (Figure 7). In the animals that received AAV2-shRNA*Pten* only, the average number of surviving RGCs was 722 ± 77/mm^2^. Partial knockout of *Sox11* in addition to *Pten* knockdown resulted in an increase in RGC survival with an average of 1393 ± 94/mm^2^ RGCs. This increased survival was statistically significant (*p* = 0.034, Mann–Whitney *U*-test, *n* = 4) (Figure 7C). The data of RGCs number in all experimental groups were summarized in Figure 8.

## Discussion

Sox family members are key regulators of neuron differentiation during development and axon regeneration after injury (Jankowski et al., 2006; Moore and Goldberg, 2011; Mu et al., 2012; Kuwajima et al., 2017; Wang et al., 2018). They are intimately involved in RGC differentiation and in the formation of the optic nerve (Chang et al., 2017). In the present study, we demonstrate that *Sox11* is upregulated following retinal injury. For RGCs, there are many known markers, such as: *Chrna6, Pou4f1, Tubb3, Thy1*, or *Sncg* (Barnstable and Drager, 1984; Gan et al., 1996; Mu et al., 2004; Surgucheva et al., 2008; Prasov and Glaser, 2012; Jiang et al., 2013; Munguba et al., 2013). Using these marker genes, we performed a meta-analysis of their expression in injured and uninjured retinas. Our injury paradigms included controlled ONC procedures that are known to induce RGC apoptosis (Hines-Beard et al., 2012; Templeton and Geisert, 2012). As expected, the expression of these RGC markers decreases as the ganglion cells die. Our data indicate an overall reduction in expression of these markers, both 2 and 5 days after retinal insult (Geisert et al., 2009). Specifically, a threefold reduction in *Sncg* expression is observed, *Chrna6* and *Pou4f1* are downregulated twofold, and, while not as robust, *Thy1* and *Tubb3* also show a decrease in expression. Collectively, these data demonstrate that known RGC markers decrease as the optic nerve degenerates.

**FIGURE 9:**
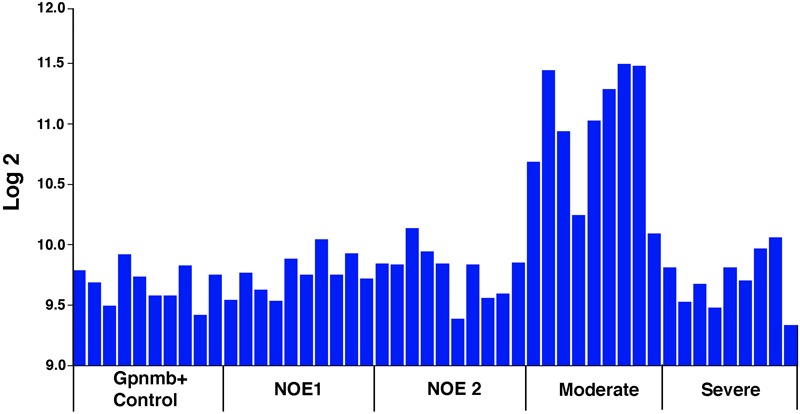
*Sox11* mRNA Levels in Pigmentary Glaucoma. *Sox11* levels in a meta-analysis of a DBA/2J mouse pigmentary glaucoma dataset from Howell et al. (2011). The ordinate represents the *Sox11* mRNA level from microarrays, expressed in log_2_, scaled with the mean set to 8. The mice were classified as wild type *Gpnmb*^+/+^ controls (Gpnmb+ Controls), no detectable glaucoma 1 (NOE 1), no detectable glaucoma 2 (NOE2), moderate glaucoma (Moderate), and severe glaucoma (Severe). Other than the wild type group, all the groups were *Gpnmb*^-/-^. Notice that there is a dramatic increase in *Sox11* expression during early phases of glaucoma in this mouse model. As the ganglion cells die in severe glaucoma, the expression levels of *Sox11* decrease. These data suggest that *Sox11* is an early marker for retinal injury. This general expression pattern was observed for the following 5 of the 6 Affymetrix probe sets targeting *Sox11*: 1429051_s_at (illustrated above), 1453002_at, 1429372_at, 1453125_at, and 1431225_at.

Unlike these RGC markers, *Sox11* levels increase substantially following the initial injury to the retina or optic nerve. Two days after ONC, there was an almost eightfold upregulation of *Sox11*. We wondered if this increase in expression was specific to the severity of retinal injury, and queried a publicly available microarray dataset of glaucomatous mice presented by Howell and colleagues as an independent test on the role of *Sox11* in the response of the retina to injury (Howell et al., 2011). In this DBA/2J mouse model of pigment dispersion glaucoma, there were relatively low levels of *Sox11* in the control mice and in mice that did not have detectable levels of ganglion cell loss (Figure 9). However, in mice with moderate ganglion cell loss, levels of *Sox11* were approximately fourfold higher than in control animals. In animals with severe glaucoma, the level of *Sox11* decreased to near control levels. These data suggest that the levels of *Sox11* decrease as the ganglion cells die. This transient upregulation of *Sox11* in moderate cases demonstrates the exquisite ability of *Sox11* to mark injured, potentially dying neurons. A similar pattern was observed following ONC in C57BL/6 and DBA/2J where *Sox11* is upregulated over twofold 2 days after nerve crush and over threefold 5 days after nerve crush (Templeton et al., 2013). Previous studies have implicated artificially high levels of *Sox11* to promote regeneration in PNS (Jankowski et al., 2009; Jing et al., 2012; Chandran et al., 2016) and corticospinal axons (Wang et al., 2015). Gene profiling showed that *Sox11* overexpression activated a set of developmental genes which are possibly related to axon growth and that it downregulated genes involved in synaptic transmission. This was consistent with the notion of a developmental process switch from axon growth mode of immature neurons to dendrite or synapse growth mode in mature neurons. Thus, *Sox11* may be regarded as a master reprogramming regulator in the nervous system. Therefore, these data confirm our hypothesis that upregulation of *Sox11* after injury is an attempt by neurons to regenerate.

What is the potential role of SOX11 following injury to the RGCs and the axons? Due to our result that injury caused upregulation of *Sox11* in different injury models, we downregulated *Sox11* 2 weeks before ONC injury by Cre/LoxP system *in vivo* in order to validate our finding. After injury, the downregulation of *Sox11* resulted in a higher survival rate of RGCs; however, no effect was seen on axon regeneration. Interestingly, we found that partially knocking out *Sox11* not only increased the survival of transduced cells but also had increased the survival rate of cells not transduced by the AAV2-Cre-GFP. External signals from microenvironment together with intrinsic signaling pathways determine whether a particular neuron will die (Pfisterer and Khodosevich, 2017). This is a clear indication that there is a cell intrinsic effect of partially knocking out *Sox11*, as can be seen in the increased survival of cells in which *Sox11* is knocked out. There is also a clear cell extrinsic effect with an increased survival of the non-transduced RGCs following the partial knockout of *Sox11*. These effects may be due to altered expression of *Sox11* downstream targets. Several studies have identified downstream targets of *Sox11* using ChIP-seq technologies (Bergsland et al., 2011; Kuo et al., 2015), including FGF2 and NGF, members of the WNT singling pathway (Ohlmann and Tamm, 2012) and TGF pathway (Garcia et al., 2017). *Sox11* is also reported to regulate the expression of BDNF (Salerno et al., 2012; Struebing et al., 2017) and GNDF (Jankowski et al., 2018), neurotropic factors known to regulate neuronal survival (Pfisterer and Khodosevich, 2017). It would not be surprising if some of these downstream targets of *Sox11* were responsible for the cell extrinsic effects associated with the partial knockout of *Sox11* in the retina.

We also observed that RGCs transduced with AAV vectors were more susceptible to ONC injury than non-transduced RGCs in the same retina. At the present time we have no direct evidence to explain this apparent difference in survival; however, it is clear that there is a decrease in the survival rate of AAV transduced RGCs. There are two potential explanations for this AAV induced susceptibility. The first is that AAV2 specifically transduces RGCs that are more susceptible to injury. Although, we do not have any direct evidence to prove this hypothesis, we do know that there is cell type selectivity in AAV transduction (Dinculescu et al., 2005; Belur et al., 2008; Watakabe et al., 2015; Smith and Chauhan, 2018). A second possibility is that the transduction by AAV-GFP itself could make RGCs be more sensitive to damage, for example, caused by some toxic effect of AAV- GFP vector (Khabou et al., 2018). It is also possible that the expression of GFP itself may result in increased susceptibility to injury (Ganini et al., 2017). Independent of the mechanism, our data reveals that the transduced cells are more susceptible to death following ONC.

In the PNS, similar response of dorsal root ganglion neurons to peripheral nerve injury was found. *Sox11* is dramatically upregulated in the dorsal root ganglion following injury to the peripheral nerve and plays a pivotal role in axonal regeneration (Jankowski et al., 2009). Three days after the transection of the sciatic nerve in the rat, there is a 1004% increase in *Sox11* in the dorsal root ganglion and these levels remain elevated for at least the next 4 days (Jankowski et al., 2009). By 4 weeks after transection, *Sox11* levels returned to baseline. This upregulation and sustained expression of *Sox11* is critical to the survival of the dorsal root ganglion neurons and the regeneration of peripheral axons along the injured nerve. When the levels of *Sox11* were knocked down by delivery of siRNA in an *in vitro* study, there was an increase in neural apoptosis as well as a decrease in neurite outgrowth (Jankowski et al., 2006). It showed that apoptosis-related genes that are regulated by *Sox11*, like *Tank, Bnip3, Blk* and *Bcl10*, may therefore contribute to the increased number of apoptotic cells in the *Sox11*-deficient cells. It seems likely that this result is not completely consistent with ours. Potential reasons include different experimental conditions (*in vivo* or *in vitro*), or different cultured cells. Jankowski showed that *In vivo, Sox11* knockdown reduced the elevation of ATF3 following PNS injury, whereas *in vitro*, this knockdown led to a decrease in ATF3 expression (Jankowski et al., 2009).

Recently, Welsbie et al. (2017) identified *Sox11*, as a major downstream mediator of RGC death through DLK/LZK signal activation. Their data with human stem cell-derived RGCs showed that after injury, primary RGCs increased *Sox11* mRNA expression. Moreover, *Sox11* knockdown had a survival-promoting effect on injured RGCs, which is consistent with our findings *in vivo*. Norsworthy et al. (2017), who were also using an *in vivo* approach, found that SOX11 is preferentially involved in the death of alpha RGCs. In addition, their data also implicated *Sox11* in axon regeneration following ONC. In our study, we further verified that downregulation of *Sox11* counters axon regeneration facilitated by *Pten* knockdown. A similar decrease in axonal regeneration occurred *in vivo* after *Sox11* knockdown in the dorsal root ganglion (Jankowski et al., 2009). Thus, the upregulation of *Sox11* is believed to be necessary for the normal regeneration of axons in the peripheral nerve. Jankowski et al. (2006) pointed out that one possible transcriptional target of SOX11, ARPC3, a protein that is thought to contribute to actin filament nucleation, may be involved in neurite extension and branching (Jankowski et al., 2006). Although further validation is required in the future, this finding still provides new insight underlying axon growth, and how the injury signal is relayed to the cell body, resulting in either cell death or regenerative response. Interestingly, *Sox11* was also upregulated when the central projection of the dorsal root was severed; however, the degree of upregulation was lower, only 51% (Jankowski et al., 2006, 2009). The most parsimonious explanation is that these neurons are attempting to survive using the same program that is successful in peripheral nerve injury; however, other influences derail the regenerative program, causing the cell to abort the regenerative process and, in many cases, cause subsequent neuronal death (Geisert et al., 1996; Schwab and Bartholdi, 1996; Silver and Miller, 2004; Harel and Strittmatter, 2006). Recent evidence indicates that these inhibitory influences can at least be partially averted by stimulating the ganglion cells to regrow their axons (Leibinger et al., 2009; Muller et al., 2009; Park et al., 2009, 2010; Smith et al., 2009; de Lima et al., 2012; Pernet et al., 2013).

**FIGURE 10:**
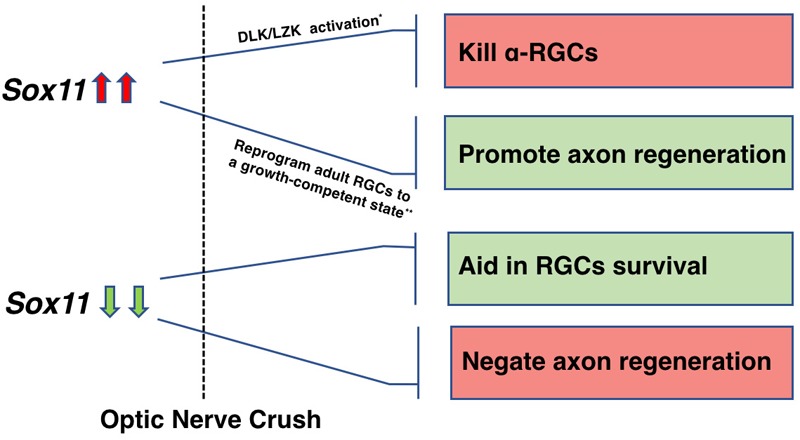
A schematic illustration depicting the role of *Sox11* regulation in RGCs survival and axon regeneration. The differential response is observed in both overexpressing *Sox11* or knocking-down *Sox11*. Upregulation of *Sox11* promotes robust axon regeneration of some RGCs types but kills α-RGCs (Norsworthy et al., 2017). Downregulation of *Sox11* aids RGCs survival (Welsbie et al., 2017) but negates the axon regeneration facilitated by *Pten* deletion.

SOX11 plays a critical role in regulating neuronal development, and has a potential promotion effect on axon regeneration. The effect of SOX11 appears to be dependent on the surrounding environment: it can either be reprogramming, or dedifferentiating (Aubert et al., 1995). Given that upregulation of *Sox11* is among the initial responses of the neuron to injury and that this response is intact in RGCs, it may be possible to identify where the transcriptional cascade leading to axon regeneration and cell survival in the CNS differs from that in peripheral nerve injury. Taken together, we found different neuron components (cell body and axons) may respond uniquely to genetic modulation. This differential response is observed in both overexpressing *Sox11* or knocking-down *Sox11*. We found that a partial knockout of *Sox11* resulted in an increase in RGC survival and a decrease in axonal regeneration; while, Norsworthy et al. (2017) found that over expressing *Sox11* had the opposite effect with a selective loss of specific RGC cell types and a facilitation of axonal regeneration (Figure 10). Therefore, in the future treatment of neural repair and axon regeneration, gene therapy is recommended to be designed specifically for multiple therapeutic targets to achieve optimal outcomes for neural recovery.

## Author Contributions

YL, FS, and EG contributed design of this study and drafted the article. JW performed all ONC procedures and data analysis. RK was responsible for animal breeding, assisted with all animal surgeries and isolated RNA from samples. YL performed intravitreal injections and immunostaining. All authors contributed to manuscript revision, read and approved the submitted version.

## Conflict of Interest Statement

The authors declare that the research was conducted in the absence of any commercial or financial relationships that could be construed as a potential conflict of interest.

## Footnotes

1 http://genenetwork.org
